# Editorial: Estimating non-monetary societal burden of livestock disease management

**DOI:** 10.3389/fvets.2025.1571267

**Published:** 2025-03-07

**Authors:** Chisoni Mumba, Guillaume Lhermie, Karl M. Rich

**Affiliations:** ^1^Department of Disease Control, School of Veterinary Medicine, University of Zambia, Lusaka, Zambia; ^2^Faculty of Veterinary Medicine, University of Calgary, Calgary, AB, Canada; ^3^Ferguson College of Agriculture, Oklahoma State University, Stillwater, OK, United States

**Keywords:** societal burden of animal diseases, socioeconomic impact of livestock diseases, one health approach, disease burden estimation, animal health policy and interventions

## Introduction

Animal diseases significantly affect various aspects of society, including agriculture, public health, and environmental sustainability. Research efforts to quantify these impacts underline the necessity of multidisciplinary approaches and evidence-based strategies to mitigate their effects. This Research Topic emphasized the need to advance our understanding of the collective burden of animal diseases through a mix of frameworks, case studies, and policy-oriented analysis.

The socioeconomic burden of disease encompasses financial costs, mortality, morbidity, and wider societal impacts. For animal diseases, this burden has predominantly been estimated using economic models that focus on monetary costs. However, such models fail to account for the significant non-monetary burden of disease, particularly in regions such as sub-Saharan Africa, where the social value of livestock often exceeds its economic value. Livestock provide resource-poor communities with food (milk, eggs and meat), agricultural benefits (draft power and manure), wealth accumulation, and cultural significance. When disease causes livestock losses, the impact reverberates through all societal levels, requiring both direct costs (market-based) and indirect costs (non-monetary) to be estimated accurately.

While direct costs can generally be quantified through market prices, indirect costs such as loss of cultural value, community status, and long-term social impacts are harder to estimate but often more consequential. These require robust mathematical and non-mathematical models for better assessment.

Despite the immense societal value of livestock, limited literature exists on metrics for estimating the non-monetary burden of livestock diseases in developing regions. Some efforts, such as the modification of Disability-adjusted Life Years (DALYs) into zDALYs, attempt to monetize the non-monetary burden using time trade-offs (1). However, such approaches have primarily been applied to zoonotic diseases that impact both humans and animals, making time trade-offs feasible. These methods remain unexplored for non-zoonotic diseases, such as East Coast fever and Contagious Bovine Pleuropneumonia, which are prevalent in sub-Saharan Africa and have substantial societal impacts.

## Key research contributions

### Quantifying and managing uncertainty

One of the key contributions to this Research Topic has been the development of robust frameworks for quantifying and managing uncertainty in animal disease burden estimation. Clough et al. presented an analytical framework that emphasizes transparency in documenting assumptions, ranking data quality, and conducting uncertainty and sensitivity analyses. Their approach emphasized the importance of acknowledging uncertainty as an integral part of the decision-making process rather than viewing it as a limitation. The proposed stepwise methodology offers a replicable model for improving the reliability of disease burden estimates and fostering stakeholder confidence in the results.

### A multisectoral perspective

Building on the need for a comprehensive understanding of the impact of animal diseases, Lysholm et al. introduced a framework for evaluating the multisectoral burden of animal diseases by integrating the impact on animal health, human health, and the environment. Their framework aligns with the “One Health” paradigm. This holistic perspective is essential for identifying interventions that maximize societal benefits while addressing the interconnectedness of health outcomes across different sectors. The authors also highlighted the role of social cost-benefit analysis in prioritizing investments and policy decisions that account for both the direct and indirect impacts of animal diseases.

### Localized case studies

The case studies featured in this Research Topic provide valuable insights into the localized impacts of animal diseases and the effectiveness of targeted interventions. Cai et al. examined the economic benefits of echinococcosis control measures in Qinghai Province, China. Their findings demonstrated the significant reduction in infection rates and economic losses achieved through dog deworming, lamb vaccination, and public education initiatives. Similarly, Kerfu et al. investigated the household-level effects of foot-and-mouth disease (FMD) in Uganda and Tanzania, revealing how market stabilization strategies and diversified livelihoods can mitigate the adverse impacts of disease outbreaks on vulnerable communities.

Oba et al. focused on the economic losses associated with respiratory and helminth infections in domestic pigs in Lira district, Northern Uganda. Their study emphasized how improving farm management practices can significantly reduce these losses, highlighting the interplay between management standards and infection control.

Zhang et al. provided a cost and revenue analysis of porcine reproductive and respiratory syndrome (PRRS) outbreaks in Chinese pig farms. They quantified the extensive economic losses caused by the disease, emphasizing the importance of effective PRRS control strategies to mitigate its impact on swine production systems.

Bessell et al. presented a high-level estimate of the net economic benefits to small-scale livestock producers arising from animal health product distribution initiatives, focusing on interventions in Africa and South Asia. Their findings underscored the transformative potential of veterinary pharmaceutical interventions in improving livelihoods and reducing the burden of disease in resource-poor communities.

### Adoption of disease control practices

Understanding the drivers and barriers to adoption of disease control practices is crucial to improving implementation and compliance. Buchan et al. provided a comprehensive review of producer perceptions regarding disease control and animal welfare practices in the dairy and beef industries. Their findings highlighted the influence of financial constraints, knowledge gaps, and stakeholder attitudes on the adoption of biosecurity measures and vaccination programs.

## Conclusion

This Research Topic has underscored the urgent need for holistic approaches to address the global burden of animal diseases. The diverse methodologies and case studies presented highlighted the critical intersection of science, policy, and practice in addressing these complex challenges by emphasizing the economic, social, and environmental dimensions of the burdens of animal diseases. These contributions lay the foundation for evidence-based interventions that promote resilience and sustainability in livestock systems.

## Future directions

The contributions to this Research Topic collectively point to the importance of integrating data-driven approaches, stakeholder engagement, and policy alignment to address the global burden of animal diseases. Moving forward, several priorities emerge:

1. Enhancing data systems: investments in data collection, integration, and accessibility are critical to improving the accuracy and reliability of burden estimates.
2. Strengthening collaboration: multisectoral partnerships are essential to address the interconnected challenges of animal, human, and environmental health.
3. Promoting equity: efforts to reduce the burden of animal diseases must prioritize the needs of marginalized and livestock-dependent communities.
4. Fostering innovation: sustainable and context-specific solutions are needed to balance economic, social, and environmental objectives.
